# Greedy knot selection algorithm for restricted cubic spline regression

**DOI:** 10.3389/fepid.2023.1283705

**Published:** 2023-12-18

**Authors:** Jo Inge Arnes, Alexander Hapfelmeier, Alexander Horsch, Tonje Braaten

**Affiliations:** ^1^ Department of Computer Science, Faculty of Science and Technology, UiT The Arctic University of Norway, Tromsø, Norway; ^2^ Institute of AI and Informatics in Medicine, TUM School of Medicine, Technical University of Munich, Munich, Germany; ^3^ Department of Community Medicine, Faculty of Health Sciences, UiT The Arctic University of Norway, Tromsø, Norway

**Keywords:** model selection, non-linear regression, prediction, restricted cubic splines, algorithm

## Abstract

Non-linear regression modeling is common in epidemiology for prediction purposes or estimating relationships between predictor and response variables. Restricted cubic spline (RCS) regression is one such method, for example, highly relevant to Cox proportional hazard regression model analysis. RCS regression uses third-order polynomials joined at knot points to model non-linear relationships. The standard approach is to place knots by a regular sequence of quantiles between the outer boundaries. A regression curve can easily be fitted to the sample using a relatively high number of knots. The problem is then overfitting, where a regression model has a good fit to the given sample but does not generalize well to other samples. A low knot count is thus preferred. However, the standard knot selection process can lead to underperformance in the sparser regions of the predictor variable, especially when using a low number of knots. It can also lead to overfitting in the denser regions. We present a simple greedy search algorithm using a backward method for knot selection that shows reduced prediction error and Bayesian information criterion scores compared to the standard knot selection process in simulation experiments. We have implemented the algorithm as part of an open-source R-package, knutar.

## 1. Introduction

Regression modeling is used in epidemiology and other fields for prediction purposes or for estimating relationships between predictor and response variables. For example, we may be interested in studying the relationship between explanatory variables and outcomes in fields such as epidemiology, biostatistics, clinical research, economics, and psychology. As a starting point, such relationships can be assumed to be linear, but when the assumption does not hold, non-linear methods can be employed. It has often been questioned if a single correct model even exists for a non-linear prediction problem (1, 2). Instead, multiple alternatives may be useful (3).

*Restricted cubic spline* (RCS) regression (4, pp. 23–26) involves partitioning the observations of a predictor variable into subintervals and piecewise fitting a third-order polynomial to each subinterval. The splines connect at join points called knots, and the RCS regression method ensures the overall function’s smoothness by forcing the first and second derivatives of the connected polynomials to agree at the knots. It additionally restricts splines to be linear in the tails of the boundary knots because unrestricted splines tend to behave poorly at the boundaries of the data (5, p. 6). RCS regression models comprise simple polynomial functions that are well-suited for interpretation by the researcher and can be combined with widely used analysis models. As Buis (6) states, “restricted cubic splines are an easy way of including an explanatory variable in a smooth non-linear way in a wide variety of models.” In epidemiology, one important use of RCS is to allow a regression coefficient, and thus the hazard ratio, in a Cox model to vary as a flexible function of time (7).

**Figure 1:**
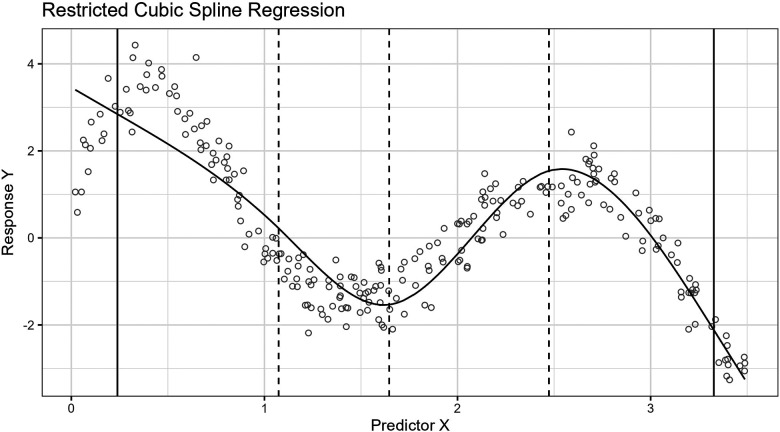
The figure shows the curve approximated by a five-knot RCS regression model fitted to a sample of 250 observations. The predictor, X, and response, Y, variables have a non-linear relationship. The inner knot locations are marked with dashed vertical lines along the horizontal axis, and the fifth and 95th percentile boundary knots are marked with solid vertical lines. The knots have been placed using the standard knot selection process, where the inner knots are placed by a regular sequence of quantiles between the boundary knots. The observations are shown as gray circles. In this example, the knots partition the observations in the sample into intervals of 56 observations, except for the lowest and highest intervals, which have 13 observations. The predictor variable is uniformly distributed, X∼U(0,3.5), and the response variable values are the sum of a fifth-degree polynomial function, X(X−1)(X−2)(X−3)(X−4), representing the true curve, and a stochastic error term, E∼N(0,0.5), representing the (homoscedastic) variance.

The standard process for placing knots for RCS regression is by a regular sequence of quantiles for the observed values of the predictor variable between two boundary knots. These boundary knots are often placed at the fifth and 95th percentiles. Recommendations for knot counts, quantiles, and boundaries are found in the study by Harrell (4, pp. 27–28). In this paper, we use the fifth and 95th percentiles as the outer boundaries for the predictor variable observations. Figure 1 shows the curve approximated by an RCS regression model fitted to a sample where the relationship between the predictor and response variable is non-linear.

Placing knots based on quantiles is an accepted convention, but there are, for example, rarely any biological or other reasons dictating that the relationship between predictor and response variables must align with equal-sized quantiles between the boundary knots. Nevertheless, there are several reasons for using a regular sequence of quantiles. For example, such quantiles are separated by the same number of observations, ensuring that observations exist between each pair of knots. In contrast, the same is not true for equidistant knots, which can lead to empty subintervals or non-convergence of the model’s fitting procedure. Further, if certain subintervals have too few observations, it can result in instability of estimates where the estimates become very sensitive to the specific values in these regions.

Using many quantiles results in knots being close to each other, especially in the denser regions of the predictor variable’s distribution. If the knots are sufficiently close, spline regressions can readily fit a model to the sample data. As the number of knots increases, the degrees of freedom and the complexity of the model increase. The problem is then the risk of overfitting, which means that with increasing model complexity, the models will often match the given sample better but not the other samples from the data-generating process or population in general. Therefore, keeping the number of knots and the respective model complexity low is desirable, yielding models that fit a given sample less exactly but generalize better. A knot count of five or less is usually considered sufficient in practice (3, 4, 8). Using five knots is a good choice when the sample size is large, n≥100, for continuous uncensored response variables, according to Harrell (4, p. 28).

Several measures for estimating the goodness of fit of a model exist that penalize higher knot counts, such as Akaike’s information criterion (AIC) and the Bayesian information criterion (BIC) (9, 10). Unfortunately, when we are limited to placing knots by a regular sequence of quantiles, having a low number of knots may miss locations essential for a good model fit. Furthermore, we risk placing knots in locations that do not substantially improve the fit or can contribute to overfitting in denser subintervals.

For spline regression models, in general, the number of knots and locations are hyperparameters that must be chosen. Perperoglou et al. (5) describe the role of spline regression models in modern biostatistics and review software packages for spline functions in R (11). The paper was written on behalf of the STRengthening Analytical Thinking for Observational Studies (STRATOS) initiative (12). The authors conclude that an experienced analyst can achieve reasonable outcomes, regardless of the spline type or tool. Most differences can be attributed to the choice of hyperparameters. However, analysts may not possess sufficient knowledge, and the availability of user-friendly, well-documented software packages for spline modeling is identified as important.

Against this background, we present a knot selection process for RCS regression models of low complexity. The process empirically shows improved results compared to placing knots separated by equal-sized quantiles for comparable knot counts. The algorithm is implemented as part of a software package for R, knutar. In addition to RCS, the function choose_model uses fractional polynomial (FP) regression (13). It selects a model based on the best goodness of fit from either FP regression, RCS with equal-sized quantiles, or RCS using the knot selection process presented in this paper.

The rest of the paper is organized as follows: Section 2 provides a brief example using real-world data, before Section 3 presents the novel knot selection process and algorithm. Section 4 describes the data generator designed for generating artificial datasets for simulation experiments. The software package with the implementation of the knot selection process and the source code repository for experiments are covered in Section 5. Section 6 describes the method for the experiments, followed by experiments and results in Section 7. The discussion is found in Section 8. Notable related work is briefly described in Section 9 before concluding in Section 10.

## 2. A brief, real-world example

Before delving into the details of the proposed knot selection process and experiments, we provide a brief example utilizing data from the Human Penguin Project (14). The purpose is to illustrate the difference between the standard knot selection process and the approach presented in the paper.

The Human Penguin Project investigates the idea of social thermoregulation in humans. It is a crowdsourced cross-national study involving 1,755 participants from various countries, and the dataset includes body temperature measurements, demographic variables, widely used psychological scales, social network indices, newly developed questionnaires, and environmental factors.

For the example, we use the age of the participants as an independent variable and the network size as a dependent variable. We then fit two models by applying RCS regression with five knots, i.e., two boundary knots and three inner knots. The inner knots for the first model are placed according to the standard knot selection process, and the inner knots for the second model are placed according to our approach.

**Figure 2:**
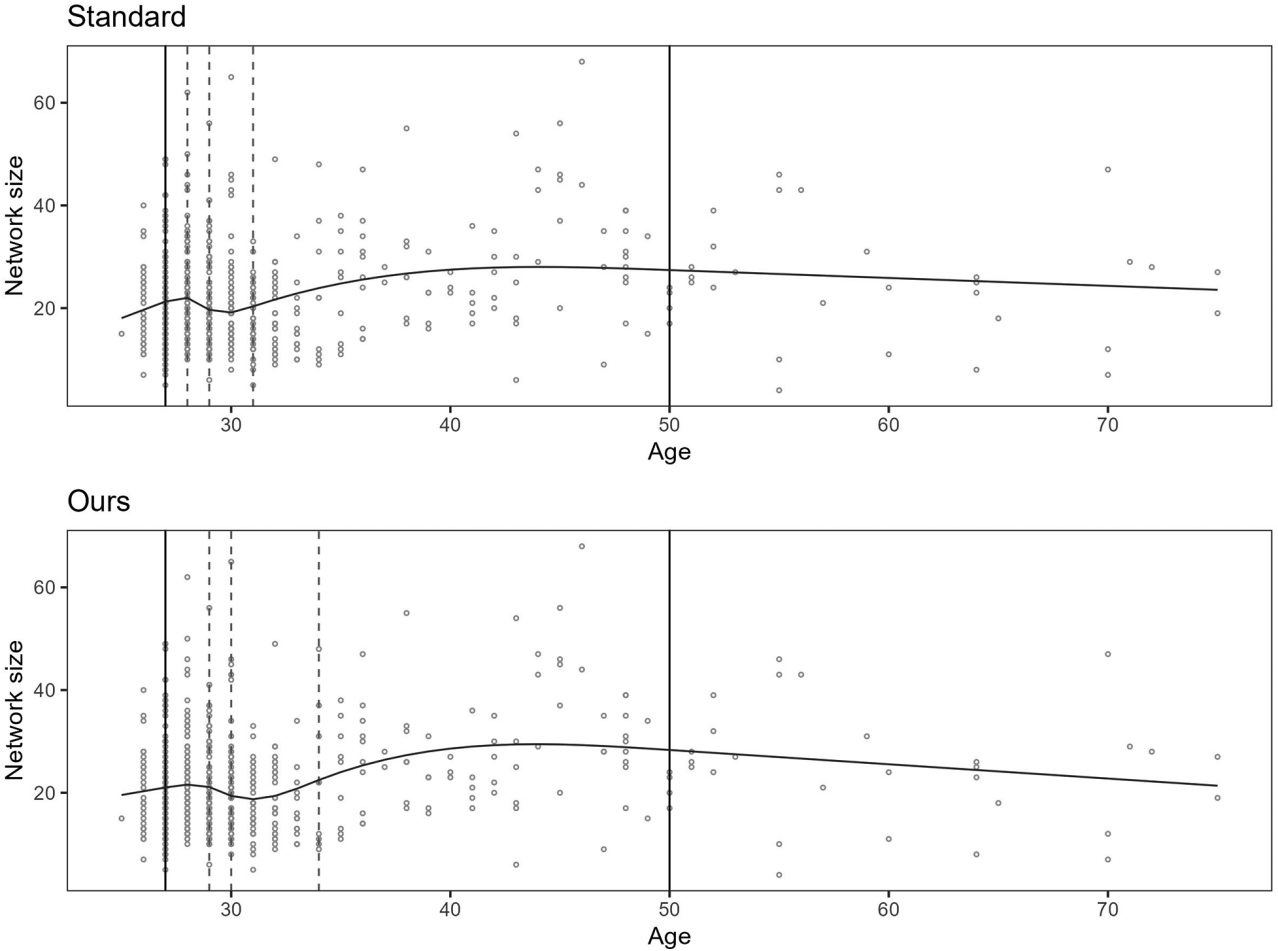
The figure shows two plots. The upper plot shows the spline for the model fitted by applying the standard knot selection process, whereas the lower plot shows the spline for our process. The observations are shown as circles, the boundary knot locations as solid vertical lines, and the inner knot locations as dashed lines. Notice that the inner knot locations and splines differ between the two plots.

The result is that the BIC scores for the fitted models are 4,368 for the standard procedure and 4,360 for ours. A lower BIC score is considered better. The splines for the models are shown in Figure 2.

## 3. Knot selection process

This section presents a process for placing knots for RCS regression. The process is an algorithm that finds a model with a good fit but an inflated number of knots and uses its knot locations to search for a less complex model. We first provide a justification of the process before describing the algorithm in more detail in Sections 3.1 and 3.2.

For the rest of the paper, we distinguish between inner and boundary knots. The *inner* knots are all knots except the two boundary knots.

The standard process for placing inner knots is by a regular sequence of quantiles between the boundaries, which leaves only a single way of placing k inner knots for a given sample. Alternatively, we could allow the inner knots to be placed freely. For freely placed knots, the number of possible ways to place k inner knots for a sample theoretically becomes infinite for a real predictor variable, x∈R. In practice, it is unnecessary to place knots indefinitely close. Beyond a certain level of precision in placing knots, the improvement in the goodness of fit obtained by further increasing the precision diminishes and becomes too small to be of practical value. Also, the precision and accuracy of the observations in the sample are generally limited, i.e., an infinite precision in placing knots is not meaningful. Consequently, there is a finite set of q locations where inner knots can be placed in the interval for the predictor variable observations. The total number of combinations that r=k inner knots can be placed for q locations is equal to the binomial coefficient:

(1) $$C(q,r)=\left(\begin{array}{c} q\\ r \end{array}\right)=\frac{q!}{r!(q-r)!}.$$

The model found by the standard knot selection process is only one in a more extensive set of possible models in the same model family. Therefore, it seems likely that other models in the same family having a better fit exist.

By increasing the distance between the candidate knot locations, the q number of locations available for knot placement becomes lower. Simultaneously, the number of possible ways to arrange the r inner knots drops substantially, as understood from the binomial coefficient. The set of locations available for knot placement can also be interpreted as a partitioning of the predictor variable interval. The knot selection process in this paper finds a reasonable partitioning of the interval where r inner knots are to be placed. It defines a manageable number of locations, q≥r, where inner knots may freely be placed and places knots so that the resulting model yields a low BIC score. In this paper, the partitioning is taken from the knot locations of a model with q≥r inner knots, often having tens of knots, fitted to the sample by applying the standard knot selection process.

The following subsections present the process as an algorithm with two main steps. The first step of the algorithm, described in Section 3.1, is to find a suitable start model. The start model’s inner knots define all the q locations where the r inner knots of a final model may possibly be placed. The second step of the algorithm starts from the full set of knot locations in the starting model and removes knot locations one by one iteratively. The aim is to find a model with r inner knots that is better than the model obtained from the standard process directly.

The strategy behind the algorithm is comparable to *backward methods* that start with complicated models, such as a high-degree polynomial, and successively simplify them (15, p. 48). It is distinct from *backward elimination* strategies that remove variables from a set of study variables (15, p. 172).

### 3.1. Finding a start model

The first step of the algorithm searches for a suitable start model by comparing the fits of a series of models up to a relatively high knot count, for example, k=0,…,50, where k is the number of inner knots. The knot count does not include the outer boundaries. A quantitative criterion for estimating the model’s goodness of fit, which additionally considers the knot count, i.e., penalizes for model complexity, is used to compare the models. AIC is one such criterion that can be used. BIC is another (9, 10). We have chosen to use BIC because it penalizes more strongly higher knot counts than AIC. Furthermore, the BIC is an asymptotically consistent model selection criterion, meaning that it almost surely, with a probability approaching one with n→∞, selects the correct model from a family containing this model (16, p. 235), where n is the sample size. For both AIC and BIC, lower scores indicate better goodness of fit, adjusted for model complexity. We select the model yielding the lowest BIC score of the assessed models as our start model.

**Figure 3:**
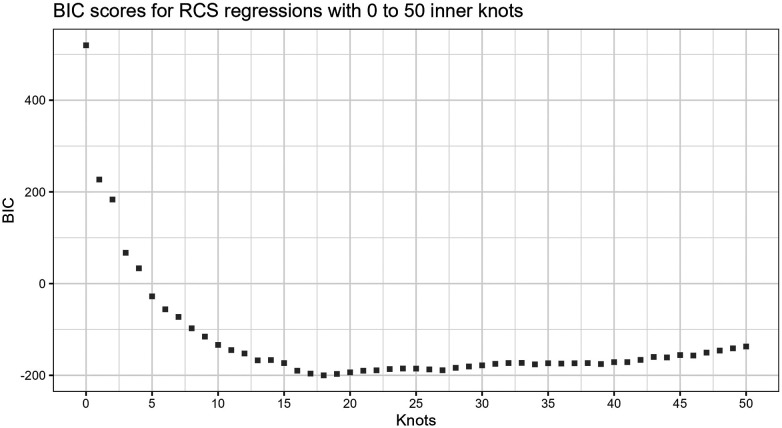
BIC scores for increasing number of knots. The lowest BIC score is at 18 inner knots in this example. The sample consists of n=250 observations with Lognormal(0.5,0.35) distribution, and the true function is cos⁡(πX). The models have fifth and 95th percentile boundary knots for the predictor variable.

Figure 3 shows the BIC scores for a set of models with different numbers of inner knots, k=0,..,50. Each model is fitted to the same sample by RCS regression and the standard procedure for knot selection. In the figure, the inner knot counts are shown along the horizontal axis, and the BIC scores along the vertical axis. The figure illustrates that increasing the number of knots typically leads to progressively lower BICs, possibly having local minima, before reaching a global minimum. Beyond this number of knots, the BIC scores increase. The curve shows how BIC reflects that overly simple or complex models have the propensity to underperform. Well-known reasons are that they may not be able to cover relations well enough or may fit to the noise, respectively. Zucchini (10) describes the former as *discrepancy due to approximation*, the latter as *discrepancy due to estimation*, and the combination as the *expected (overall) discrepancy*. The discrepancy due to estimation increases as the number of knots increases. This may be less severe for larger samples, e.g., having thousands of observations. In that case, the increase in overall discrepancy becomes less steep. In this paper, the focus is on hundreds of observations per sample rather than thousands.

Given the low BIC, the start model has a relatively low expected (overall) discrepancy. However, it has far more knots than the maximum we want for the final model. It seems probable that the start model is overfitted but that a subset of the knot locations can be a good choice for building a less complex model.

### 3.2. Lowering the model complexity

The start model has the lowest BIC score of all assessed models found by applying the standard knot selection process for a range of knot counts and, presumably, a low expected (overall) discrepancy. Next, the second step of the algorithm uses this model as a starting point for iteratively searching for a less complex model while keeping the BIC low.

Let q be the number of inner knots in the start model, and r be the target knot count of the less complex model. The value of q depends on the first step of the algorithm, and r is decided up front by the analyst and fixed. Let S be the set of locations of the inner knots in the start model.

A possible approach for finding a less complex model is to compare all possible models having inner knots at r locations selected from S. We can then perform an exhaustive, brute-force search and be guaranteed to find the best model in this candidate set, B. Unfortunately, the size of B rapidly grows as q increases. We observe that the number of models in the set equals the binomial coefficient, |B|=C(q,r) (Equation 1), meaning that an exhaustive search does not scale well from a computational standpoint. In Θ-notation (17, pp. 48–49), it has a factorial time complexity, Θ(q!), holding r fixed.

Here, we present a *greedy algorithm* (17) that starts with the complete set of knot locations from the start model, S, selected. It then removes knot locations one by one. The algorithm is a state space search (18, p. 67) using a simple heuristic: Identify and remove the knot location with the least undesirable impact on the BIC score when removed. This knot location is deemed the most redundant in the current set, which implies an assumption that the individual knot locations in a model can be ordered by their relative contribution to a good fit, from being crucial to redundant. Note that it only matters which knot location is the *most* redundant for each iteration step. Only the most redundant knot location is removed. As long as none of the r knot locations for the globally best model in B have been ranked as the *most* redundant in an iteration step, the found model will be identical to the globally best model in B. When r=q−1, the state space search algorithm is equivalent to assessing all models in B. Otherwise, the resulting final model may differ from the best model in B. Thus, the algorithm does not guarantee finding the best model in B. However, it is relatively common for state space search, or machine learning algorithms in general, not to guarantee a globally optimal solution.

In the first iteration step, the algorithm assesses which one of the start model’s q inner knot locations to remove first. For this, it assesses q candidate models. In the next step, the algorithm assesses which one of the q−1 inner knot locations of the model found in the previous step to remove next, which requires the assessment of q−1 candidates. The iteration continues until r inner knot locations are left. For the last step, r+1 models are assessed. Thus, the total number of models assessed by the algorithm is the sum of natural numbers from r+1 to q inclusive. Here, we assume that r<q. By applying Equation 2 for a=r+1 and b=q and expanding, we get Equation 3.

(2) $$\sum_{\;j=a}^{b}j\equiv \frac{\left(a+b)(b-a+1\right)}{2},$$

(3) $$\sum_{\;j=r+1}^{q}j\equiv \frac{q+(r+1)}{2}\left(q-r\right)\equiv \frac{1}{2}(q^{2}+q-r^{2}-r).$$

From the right-hand side of Equation 3, we see that the time complexity is quadradic, $\Theta (q^{2})$, holding r fixed. From Equations 1 and 3, we see that for r≤2, the exhaustive search requires fewer models to be assessed than the state space search algorithm. The two approaches are equivalent when r=q−1. Otherwise, the number of models assessed by the exhaustive search grows far more rapidly with increasing q. For example, if the start model has q=30 inner knots and the final model has r=4 inner knots, the exhaustive search assesses 27,405 models. The version that removes one knot at a time only assesses 455 models. This difference is monotonically and steeply increasing as q increases. Further, instead of allowing only a specific number of knots for the final model, we can accept a final model having a knot count in a given range. When assessing a sequence of allowed number of inner knots, $k=0,\ldots ,k_{\max}$, removing one knot at a time is computationally cheaper because the exhaustive search requires the complete set of possible models per target knot count, $B_{k},k=0,\ldots ,k_{\max}$, for each step. In conclusion, the state space search scales better computationally. A combination of the exhaustive and the state space search can be used. For example, the exhaustive search can find the best model with regard to BIC in B when r≤2. However, the experiments described in Sections 6 and 7 only use the state space search because it is the paper’s primary focus.

We may be tempted to remove all but the r most crucial inner knots in one step, but this approach can be suboptimal. When a single knot is removed, the order of the remaining inner knots by relative importance can change. A hypothetical example is when two or more inner knots are clustered around a location crucial to a good fit, e.g., an essential critical point. Individually removing any of these inner knots may have a low negative impact because the other inner knots still support the shape of the curve. On the other hand, if we remove all these inner knots, no knots would contribute to the shape of the curve around the crucial location. The consequence would be a significant negative impact on the goodness of fit. When we remove knots one by one, we allow the order of the knots by relative importance to change per iteration step. For example, if only one of the knots around a crucial location is still present in the set, its importance will be ranked as high, preventing it from being removed.

Finally, we could also imagine a different greedy algorithm that starts with no inner knots and iteratively adds knots at locations selected from S. Each iteration step adds a new knot by selecting, from S, the knot location that yields the best model of the alternatives. When the algorithm selects the first knot location and fits a model with a single inner knot, the discrepancy due to approximation can be expected to be high in many cases. Thus, the selected knot location may not be essential to recreate the start model’s basic regression curve shape. Unfortunately, the algorithm will not replace knot locations in subsequent iteration steps, meaning new knots can be placed based on misselected locations from the early steps.

### 3.3. Accepting a range of knot counts

In the previous section (Section 3.2), we described the algorithm as targeting one specific number of knots for the final model. However, we do not target only one specific number of knots in the software package (Section 5), experiment methods (Section 6), and experiments (Section 7). Instead, the final knot count is allowed to be within a range, and the model yielding the best BIC score in that range is selected as the final model. We also do the same for the standard knot selection process and select the model having the best BIC score within the given range of inner knot counts, $k=0,\ldots ,k_{\max}$.

To find a final model having an inner knot count within a target range $k=0,\ldots ,k_{\max}$ using the algorithm presented in this paper, the iteration described in Section 3.2 continues until all inner knots have been removed. The two boundary knots are never removed. The algorithm selects the best of the models found during the last iteration steps, where $k\leq k_{\max}$, as the final model.

### 3.4. Knot removal example

**Figure 4:**
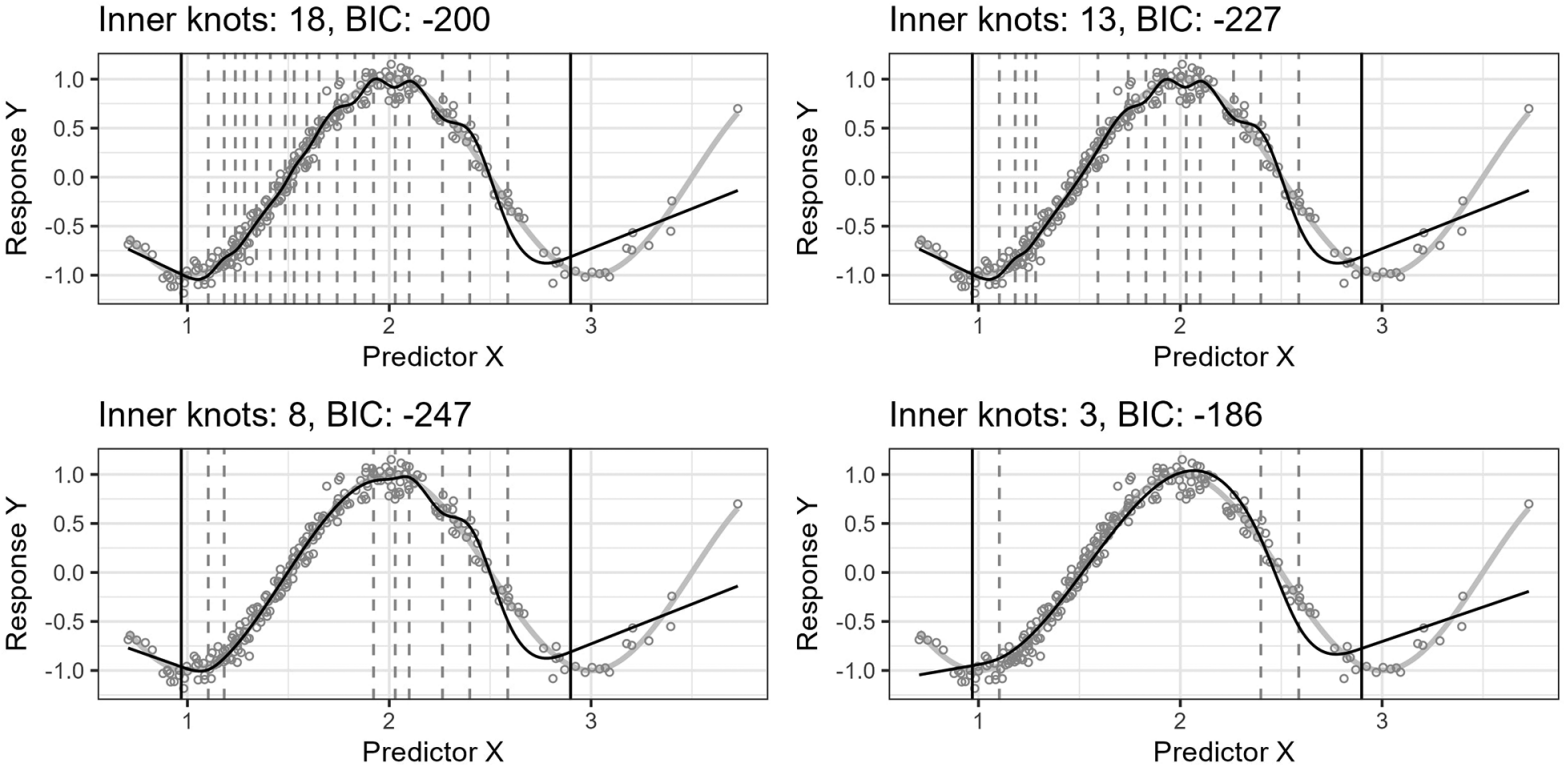
The figure shows the effect of iteratively removing knot locations one by one from the start model. In each of the four plots, the thin, black line is the model’s fitted curve, whereas the thicker, light gray line is the true curve. The smaller circles are the observations. The inner knot locations are shown as dashed vertical lines, whereas the fifth and 95th percentile boundary knot locations are shown as solid, black vertical lines.

Figure 4 shows the effect of the algorithm iteratively removing knot locations from the start model in the search for a final model of lower complexity. The resulting regression curves at four different steps are plotted. The predictor variable is lognormally distributed, X∼Lognormal(0.5,0.35), and the true curve is a cosine function, cos⁡(πX). The algorithm finds a start model by applying the standard knot selection process for a range of inner knot counts, k=0,…,50. The model at 18 inner knots yields the best BIC for this sample. The algorithm then systematically removes knot locations from this start model. At each iteration step, the algorithm assesses all models with j of j+1 inner knot locations from the previous step, i.e., j+1 models, and selects the model yielding the lowest BIC score. The figure shows the best models for 18, 13, 8, and 3 inner knots, having BIC scores of −200, −227, −247, and −186, respectively. Each step does not necessarily have a lower BIC score than the previous step because the BIC score can increase as the number of knots decreases because of discrepancy due to approximation (underfitting).

Notice that the start model with 18 inner knots in Figure 4 has many redundant knots. As the algorithm removes such knots, the basic shape of the regression curve stays relatively stable for this example. Also, the start model’s curve has some wiggliness around the top turning point. It is an example of overfitting in a region where the distribution of the observations for X is denser and illustrates one problem of placing knots by a regular sequence of quantiles.

**Figure 5:**
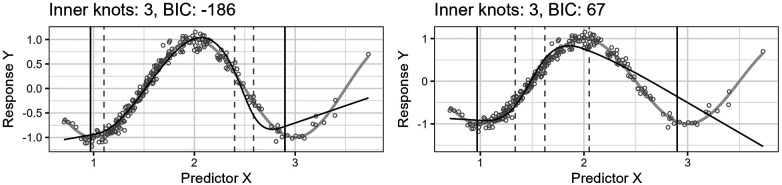
The left plot shows the curve for the final model in Figure 4. The right plot shows the curve for the model obtained by using the standard knot selection process for three inner knots directly.

Figure 5 shows the predicted curve for the final model in Figure 4 and the corresponding model obtained by applying the standard knot selection process for three inner knots directly, yielding a BIC of 67. Notice that the observations are more sparsely distributed for greater values of X, making the upper quantile before the boundary in the right-side plot of Figure 5 wider and the model underfitted. This underfitting illustrates another problem concerning the standard knot selection process.

## 4. Data generator

A data generator was designed and implemented for generating the artificial datasets used in the experiments. It produces pseudo-random samples by applying three user-defined functions:

1. The distribution X of the predictor variable $X=\{x_{1},\ldots ,x_{n}\}∼X_{n}$, where n is the sample size. For example, the predictor variable distribution can be X=Lognormal(μ,σ).
2. The ground truth function, ϕ, for generating the population means, F, for the response variable Y given X. The term *true curve* is used in the paper for these population means (Equation 4).
  (4) $$F=\mu_{Y|X}=\phi (X).$$
3. The distribution for the error component, E, around $\mu_{Y|X}$. For example, it can be a normal distribution (Equation 5).
  (5) $$E∼N(\mu_{E},\sigma_{E}).$$

First, the data generator draws a sample of n predictor variable observations, $X∼X_{n}$. For repeatability, the user can optionally set the seed used internally by the pseudo-random number generator. Next, the generator computes the true curve values, F=ϕ(X). The generator then computes the response variable values, $Y=F+(E∼E_{n})$. The error component distribution E is user-defined and can alternatively be heteroscedastic. For example, we can scale the error distribution’s variance by a factor of the given value of x∈X, which can be relevant for ratio-valued variables. Finally, the sample values can be rounded to a chosen accuracy, simulating the limits of the measurement method. The resulting dataset includes both the rounded and unrounded values. The simulation experiments in this paper only use homoscedasticity and unrounded values to avoid unnecessary complexity.

X and Y are the input predictor and response variable values used for fitting the model, whereas F is the ground truth values used to assess the performance of the model’s predictions in the experiments. Together, they form the variable Z having the distribution $Z_{n}$ (Equation 6).

(6) $$Z=(F,X,Y)∼Z_{n}.$$

## 5. Software package implementation

The implementation of the knot selection algorithm presented in this paper is included as part of our package, knutar, for use with R (11). The package is publicly available at https://github.com/jo-inge-arnes/knutar and contains functions for suggesting models and utility and plotting functions. It also includes the function generate_data for generating artificial datasets.

The function choose_model in the package assesses different regression models from a set of regression methods, returning the one yielding the best results according to an information criterion, where the default information criterion is BIC.

The function’s strategy for choosing the appropriate regression model follows a forward method going from simple to more complicated methods. The knutar package uses the standard generalized linear regression models (GLM) function in R, stats::glm, for building models. It first applies regressions with multivariable fractional polynomials, mfp (19), which internally uses a forward selection process for fractional polynomials (13) that includes simple linear regression. Next, the function uses RCS regression, splines::ns, and finds the number of knots, $k\leq k_{\max}$, yielding the best score for equal-sized quantiles. Lastly, the function applies the knot selection process presented in the paper. The model with the best information criterion score is returned along with extra information. If models from different methods give the same best score, the function chooses the model stemming from the earliest of the applied methods. The function additionally returns a list with the best candidate model from each of the three regression methods and information about the chosen hyperparameters.

The main parameters of choose_model are the dataset for the sample, the response variable, and the predictor variable(s). The function uses the response and predictor variables as the left- and right-hand sides of a formula so that the predictor variable can be a formula composed of one or more of the variables available in the dataset. In addition, choose_model provides optional input parameters to replace the default information criterion, maximum number of knots, and more.

The R-scripts for running the paper’s accompanying experiments are in a separate repository at https://github.com/jo-inge-arnes/knutar-experiments.

## 6. Methods

We conduct four simulation experiments as application examples of different functions, ϕ, for the true curve. The design of the simulation experiments follows the theoretical framework for inference problems in benchmark experiments presented in the study by Hothorn et al. (20).

### 6.1. Simulation experiment

Using the data generator described in Section 4, artificial observations are generated by drawing from known distributions. The ground truth is known. Each $z\in Z∼Z_{n}$ consists of the value for the predictor variable, x, the response variable, y, and the ground truth that is to be predicted, $f=\mu_{y|x}$ (Equation 7).

(7) z=(f,x,y).

For each simulation experiment, we generate artificial data with a defined distribution, Z, and draw a set of M=1,000 learning samples consisting of n=250 observations (Equation 8).

(8) $$L^{1},\ldots ,L^{M}∼Z_{n}.$$

Two algorithms, $a_{1}$ and $a_{2}$, are compared in the experiments, each yielding a single fitted model per learning sample with a maximum of three inner knots, $k_{\max}=3$:

• $a_{1}$ fits models having $k=0,\ldots ,k_{\max}$ inner knots to the given learning sample by using the standard equal-sized quantiles approach and selects the model yielding the lowest BIC score.
• $a_{2}$ uses the knot selection process presented in this paper and selects the model with an inner knot count in the range $k=0,\ldots ,k_{\max}$ that yields the lowest BIC score.

By pairwise applying $a_{1}$ and $a_{2}$ to each of the m learning samples, we get the fitted models (Equation 9).

(9) $$a_{im}=a_{i}(\cdot |L^{m}),\;i=1,2.$$

The fitting procedures for the algorithms are deterministic, meaning they do not depend on random starting values or hyperparameters outside the learning samples. Also, the finished models, $a_{im}$, no longer depend on hyperparameters. Further, the models are themselves random variables depending on $L^{m}$ and have a distribution dependent on the data-generating process (Equation 10).

(10) $$a_{im}∼A_{i}(Z_{n}).$$

The model performances are measured with a scalar function, p, which can also be interpreted as a random variable with a distribution dependent on the data-generating process (Equation 11).

(11) $$p_{im}=p(a_{i},L^{m})=p(a_{im})∼P_{i}=P(Z_{n}).$$

For each model-pair, $a_{1m}$ and $a_{2m}$, fitted per learning sample, $L^{m}$, we draw t=2,000 observations from the same data-generating process as the learning samples, z=(f,x,y)∈T, where $T∼Z_{t}$. However, because predictions for RCS models are most reliable between the boundary knots, we ensure that the x-values are within this range.

The performance per model, ${\hat{\;p}}_{im}$, is computed by approximating the expected loss between the ground truth, f=ϕ(x), and the predicted value, $\hat{y}=a_{im}(x)$ (Equation 12).

(12) $${\hat{\;p}}_{im}=\hat{\;p}(a_{i},L^{m})=\frac{1}{t}\sum_{z=(f,x,y)\in T}L(f,\hat{y}),$$

where L is the following quadratic loss function (Equation 13).

(13) $$L(f,\hat{y})=(f-\hat{y}{)}^{2}.$$

This gives us two random samples consisting of M approximated performance measure values from the distributions $P_{1}(Z_{n})$ and $P_{2}(Z_{n})$, one set for each algorithm. We now formulate the null hypothesis, where ${\hat{P}}_{i}$ is the approximation of $P_{i}$. The null hypothesis is rejected at a significance level of α=0.05 (Equation 14).

(14) $$H_{0}\;:\;E({\hat{P}}_{1}(Z_{n}))=E({\hat{P}}_{2}(Z_{n})).$$

Because the models for $a_{1}$ and $a_{2}$ are fitted to the same learning sets, the natural experimental design is a paired K samples design, as described in Section 4 of Hothorn et al. (20). The paired difference test t-statistic is used under the null hypothesis of equality of the performance measure distributions.

### 6.2. Comparing BIC scores

In addition to hypothesis testing the distributions of the estimated performance measure, we also report the differences in BIC scores between $a_{1}$ and $a_{2}$, as well as the sample mean difference in knot counts. These are meant as descriptive, whereas the main hypothesis is on the performance measure as described in the previous subsection.

## 7. Experiments and results

For the application example experiments, we followed the method described in Section 6. Four different non-linear functions representing true curves were defined, F=ϕ(X). A lognormal distribution was used as X (Equation 15) for the experiments in Sections 7.2–7.4. For the experiment in Section 7.5, the distribution was uniform (Equation 16). The error component distribution, E, was homoscedastic and normal (Equation 17) for all experiments. The values for the true curve, F, and error component, E, were added to obtain the values for Y (Equation 18). The fifth and 95th percentiles were used as lower and upper boundaries for the X observations for the samples. For the t=2,000 observations, z=(f,x,y)∈T, used to estimate the performance measure, ${\hat{\;p}}_{im}$, all values were between these boundaries. The reason is that RCS models are not reliable outside the interval of the predictor variable observations used to fit a model.

(15) X∼Lognormal(0.5,0.35),

(16) X∼U(0,3.5),

(17) E∼N(0,0.1),

(18) Y=F+E.

### 7.1. Result report structure

**Figure 6:**
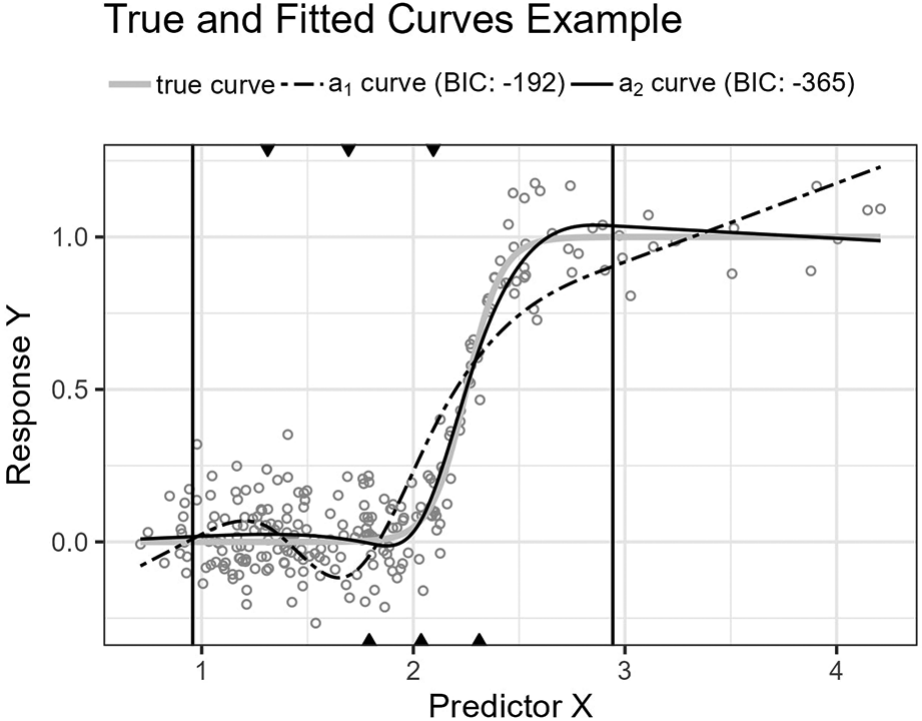
The figure shows the logistic function’s curve together with the curves approximated by $a_{1}$ and $a_{2}$ for an example sample of 250 observations. The vertical lines are the outer fifth and 95th percentile boundaries. The downward-pointing triangles along the top horizontal axis mark the inner knot locations for $a_{1}$, and the upward-pointing triangles along the bottom axis are the knot locations for $a_{2}$.

**Figure 7:**
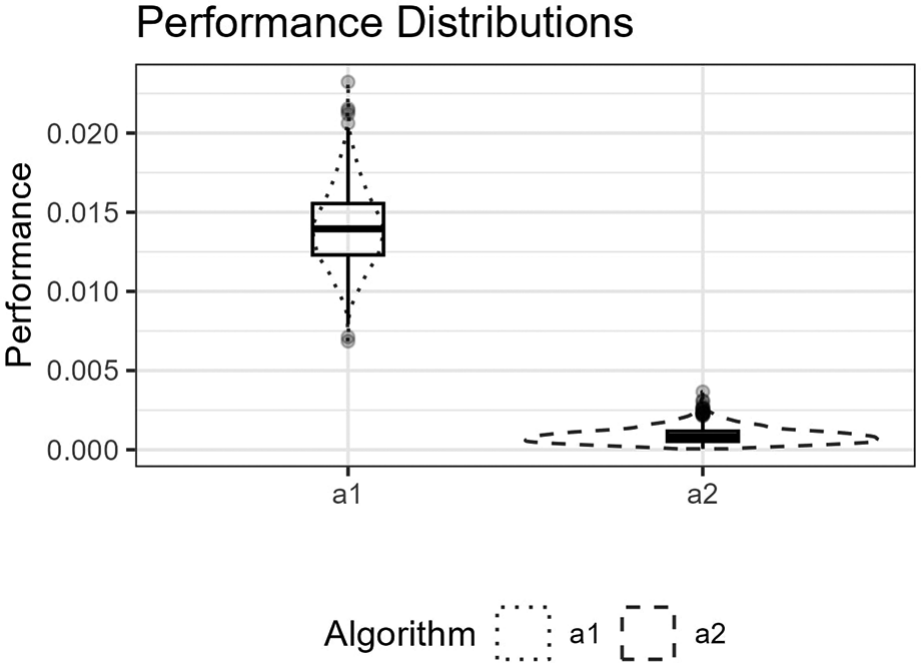
The figure shows the distributions for $\hat{\;p}(a_{1},L^{m})$ and $\hat{\;p}(a_{2},L^{m})$ for the logistic function as box plots within violin plots.

**Figure 8:**
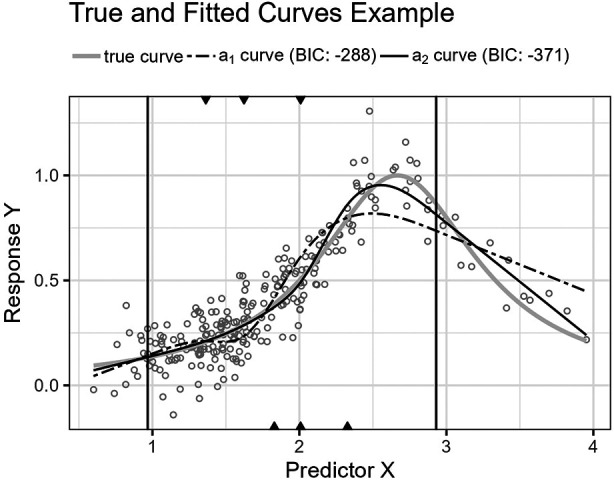
The figure shows the Runge function’s curve together with the curves approximated by $a_{1}$ and $a_{2}$ for an example sample of 250 observations. The vertical lines are the outer fifth and 95th percentile boundaries. The downward-pointing triangles along the top horizontal axis mark the inner knot locations for $a_{1}$, and the upward-pointing triangles along the bottom axis are the knot locations for $a_{2}$.

**Figure 9:**
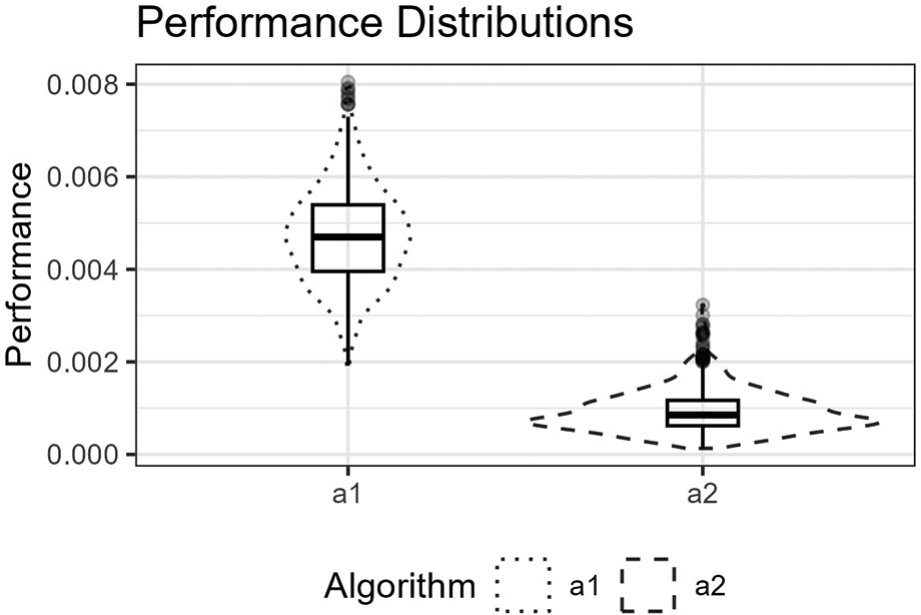
The figure shows the distributions for $\hat{\;p}(a_{1},L^{m})$ and $\hat{\;p}(a_{2},L^{m})$ for the Runge function as box plots within violin plots.

**Figure 10:**
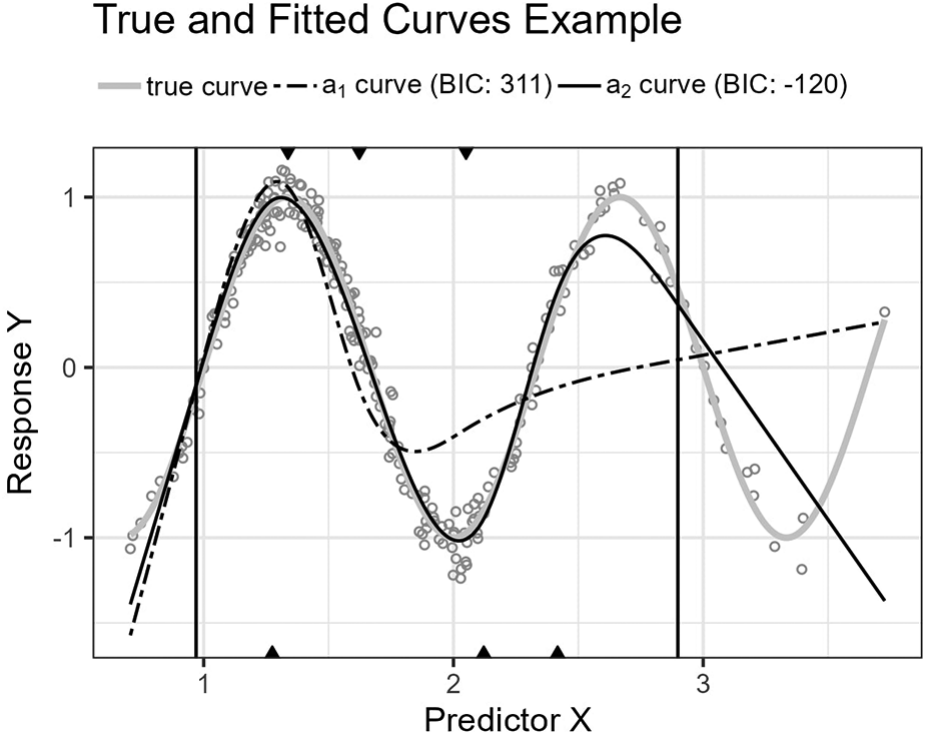
The figure shows the trigonometric function’s curve together with the curves approximated by $a_{1}$ and $a_{2}$ for an example sample of 250 observations. The vertical lines are the outer fifth and 95th percentile boundaries. The downward-pointing triangles along the top horizontal axis mark the inner knot locations for $a_{1}$, and the upward-pointing triangles along the bottom axis are the knot locations for $a_{2}$.

**Figure 11:**
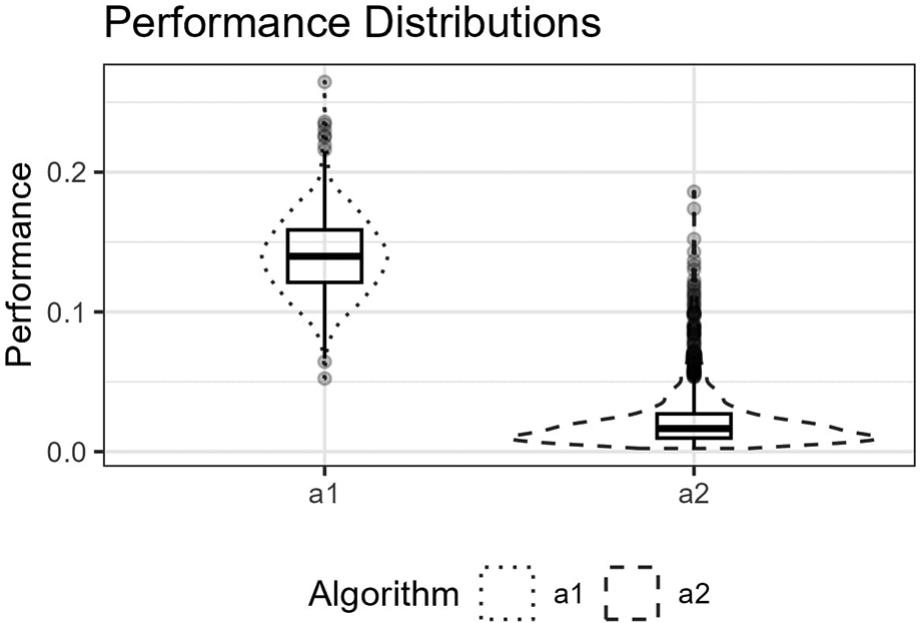
The figure shows the distributions for $\hat{\;p}(a_{1},L^{m})$ and $\hat{\;p}(a_{2},L^{m})$ for the trigonometric function as box plots within violin plots.

**Figure 12:**
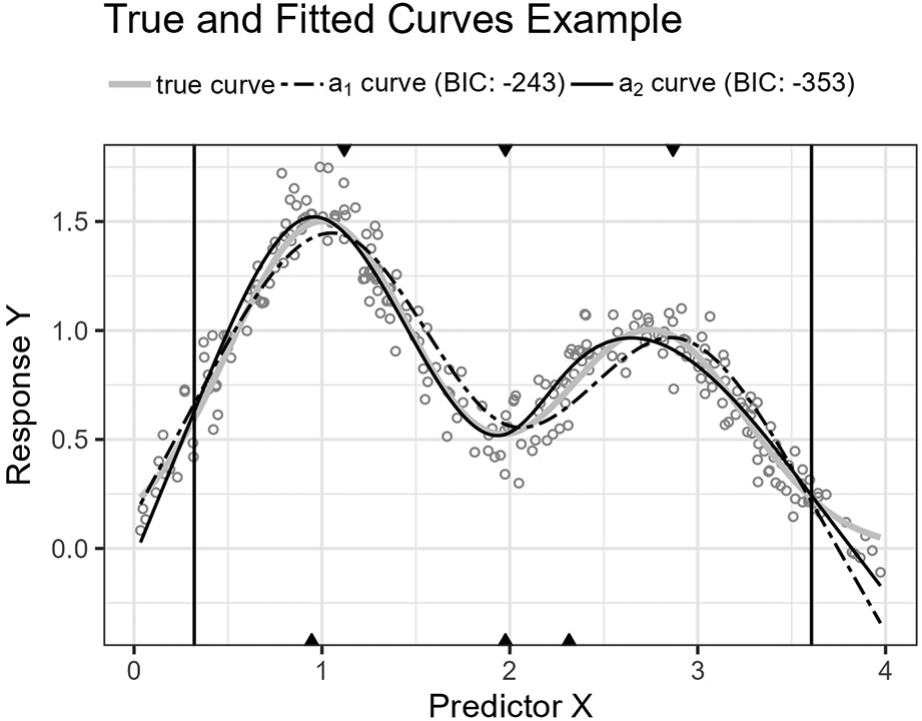
The figure shows the Gaussians function’s curve together with the curves approximated by $a_{1}$ and $a_{2}$ for an example sample of 250 observations. The vertical lines are the outer fifth and 95th percentile boundaries. The downward-pointing triangles along the top horizontal axis mark the inner knot locations for $a_{1}$, and the upward-pointing triangles along the bottom axis are the knot locations for $a_{2}$.

**Figure 13:**
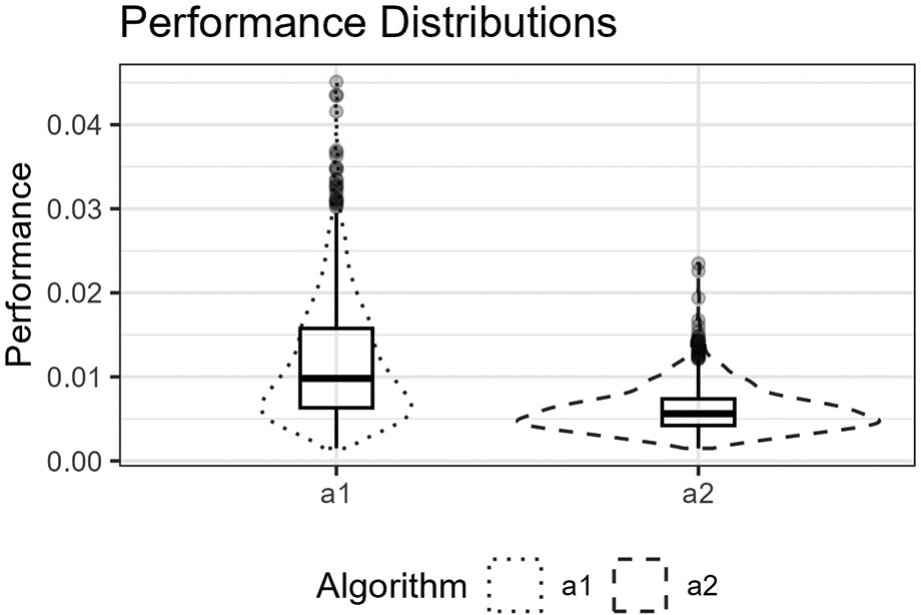
The figure shows the distributions for $\hat{\;p}(a_{1},L^{m})$ and $\hat{\;p}(a_{2},L^{m})$ for the Gaussians function as box plots within violin plots.

The results from the two-tailed paired sample t-tests for the differences in estimated performance measures and BIC scores for the four experiments are reported together in Table 1. Section 7.6 describes the table columns. The functions representing the true curves and figures illustrating the results are described in four subsections following the same structure (see Sections 7.2–7.5):

1. The experiment’s ground truth curve function, $\phi_{i}$, is briefly described and the formula defined see Equations 19–22.
2. The function description is followed by a plot showing an example of the resulting true and fitted curves for a single sample. The BIC scores for the fitted curves are included in the plot’s legend see Figures 6, 8, 10, and 12.
3. A figure showing the distributions of the estimated performance measure, ${\hat{\;p}}_{im}$, for $a_{1}$ and $a_{2}$ as boxplots within violin plots see Figures 7, 9, 11, and 13.

**Table 1:** Experiment results.

Curve	$E({\hat{P}}_{1})-E({\hat{P}}_{2})$ CI	${\bar{P}}_{1}$	${\bar{P}}_{2}$	$E(\mathrm{BIC}(a_{1m})-\mathrm{BIC}(a_{2m}))$ CI	${\bar{d}}_{k}$
Logistic	[$1.295\times 10^{-02}$, $1.326\times 10^{-02}$]	$1.401\times 10^{-02}$	$9.086\times 10^{-04}$	[198, 203]	0.0
Runge	[$3.721\times 10^{-03}$, $3.848\times 10^{-03}$]	$4.724\times 10^{-03}$	$9.391\times 10^{-04}$	[78, 81]	0.2
Trigonometric	[$1.144\times 10^{-01}$, $1.186\times 10^{-01}$]	$1.401\times 10^{-01}$	$2.354\times 10^{-02}$	[244, 258]	0.0
Gaussians	[$5.187\times 10^{-03}$, $6.105\times 10^{-03}$]	$1.175\times 10^{-02}$	$6.102\times 10^{-03}$	[59, 69]	0.0

### 7.2. Logistic

The first curve function, Equation 19, is a logistic function. Such functions are seen in many fields, including sigmoid activation functions for artificial neural networks (16, p. 392) and logistic regression (15, p. 682). However, logistic functions are also encountered in the context of non-linear relationships between predictor and response variables, where population growth is one example (21, p. 390).

Figure 6 shows the function’s curve and the curves approximated by $a_{1}$ and $a_{2}$ for an example sample of 250 observations. Figure 7 shows the distributions of the estimated performance measure for $a_{1}$ and $a_{2}$ as box plots within violin plots.

(19) $$\phi_{1}(X)=(1+e^{-12X+27}{)}^{-1}.$$

### 7.3. Runge

The second function, Equation 20, is a Runge function. Such functions are commonly used to demonstrate Runge’s phenomenon where fitting high-order polynomials by equidistant interpolation points results in oscillation at the endpoints and failure to converge (22, p. 101). Here, the curve is translated so that the central peak is at $X=\frac{8}{3}$. We do not use high-order polynomials, but lower-order fractional polynomials also perform worse than splines for this function.

Figure 8 shows the function’s curve and the curves approximated by $a_{1}$ and $a_{2}$ for an example sample of 250 observations. Figure 9 shows the distributions of the estimated performance measure for $a_{1}$ and $a_{2}$ as box plots within violin plots.

(20) $$\phi_{2}(X)=\frac{1}{1+(\frac{3}{2}X-4{)}^{2}}.$$

### 7.4. Trigonometric

The third function, Equation 21, is a cosine function. The function’s turning points require an economic placement of the three available inner knots.

Figure 10 shows the function’s curve and the curves approximated by $a_{1}$ and $a_{2}$ for an example sample of 250 observations. Figure 11 shows the distributions of the estimated performance measure for $a_{1}$ and $a_{2}$ as box plots within violin plots.

(21) $$\phi_{3}(X)=\cos\left(\frac{3\pi }{2}X\right).$$

### 7.5. Gaussians

The last curve function, Equation 22, is the sum of two Gaussian functions (Equation 23) reminiscent of a bimodal distribution with two normal distributions, $\frac{3}{2}N(1,0.5)$ and N(2.75,0.5). As the only experiment, X has a uniform distribution, X∼U(0,3.5), in contrast to the other three experiments that use a lognormal distribution.

Figure 12 shows the function’s curve and the curves approximated by $a_{1}$ and $a_{2}$ for an example sample of 250 observations. Figure 13 shows the distributions of the estimated performance measure for $a_{1}$ and $a_{2}$ as box plots within violin plots.

(22) $$\phi_{4}(X)=\frac{3}{2}\gamma \left(X,1,\frac{1}{2}\right)+\gamma \left(X,\frac{11}{4},\frac{1}{2}\right),$$

where γ(X,a,b) is the following Gaussian function,

(23) $$\gamma (X,\mu ,\sigma )=\frac{1}{\sigma \sqrt{2\pi }}\exp\left(-\frac{1}{2}\frac{(X-\mu {)}^{2}}{\sigma^{2}}\right).$$

### 7.6. Results table

Table 1 shows the results for the four experiments described in Sections 7.2–7.5. The columns are

1. The function representing the true curve in the experiment,
2. The 95% confidence interval for the expected difference in estimated performance measure for $a_{1}$ and $a_{2}$,
3. The performance measure sample mean for $a_{1}$,
4. The performance measure sample mean for $a_{2}$,
5. The 95% confidence interval for the expected difference in BIC scores for $a_{1}$ and $a_{2}$,
6. The sample mean for the number of knots for $a_{1}$ minus the number of knots for $a_{2}$.

## 8. Discussion

The implementation of the knot selection algorithm presented in this paper is part of an R-package, knutar. The package-function choose_model uses the algorithm but only selects the model produced if it scores better than the models from fractional polynomial regression and the standard knot selection process for RCS. Thus, we do not intend the presented process to replace the standard knot selection process but as an option in addition to it. The results can often be similar, so the processes agree. However, as the experiments show, there are cases where the models produced by our knot selection process perform significantly better.

For all four experiments, Section 7.2–7.5, the null hypothesis, Equation 14, was rejected. We do not report the exact p values in the results table. The reason is that we can, in principle, generate infinitely many artificial test observations, and the p value will reach zero in the limiting case when the two distributions are different. We can detect performance differences with high power. However, as discussed by Hothorn et al. (20, p. 697), “one should always keep in mind that statistical significance does not imply a practically relevant discrepancy and therefore the amount of the difference should be inspected by confidence intervals and judged in the light of analytic expertise.” Instead, we can turn to the presented confidence intervals to inspect the amount of difference. The expected difference in BIC scores for $a_{1}$ and $a_{2}$ reported in Table 1 suggests that our knot selection process achieves a clear improvement compared to the standard process for the example experiments.

Predictor variables usually have non-uniform distribution for real data. When equal-sized quantiles separate knots, more knots are located in dense regions than in sparser regions of the predictor variable. Suppose the number of knots is relatively low compared to the non-linear curve shape for the relationship between the predictor and response variables. Placing inner knots by a regular sequence of quantiles may lead to too few knots and underperformance in the sparser regions, i.e., discrepancy due to approximation, which is the case for the right-side regions of Figures 6, 8, and 10. In these regions, our knot selection process shows a better adaptation to the true curve. In Figure 10, we see that there are more critical points than in Figures 6 and 8. In such cases, the selected locations for the few available knots become more crucial to the resulting goodness of fit.

Conversely, the standard knot selection process can lead to overfitting in denser regions. For example, in Figure 6 and 8, we see that the curve for $a_{1}$ is wiggly compared to the true curve, indicating an overfitted $a_{1}$ curve.

The experiment in Section 7.5 uses a uniform distribution for X. Therefore, the density of the predictor variable observations does not systematically differ in any particular region of the sample for the M=1,000 learning samples. However, the ground truth curve for the experiment has a bimodal shape with turning points that do not necessarily align well with a few knots distanced by equal-sized quantiles. Here, the presented selection process has greater flexibility in placing knots and can better fit a regression spline using the same number of knots.

RCS models are often unreliable in the tails, i.e., before the first boundary knot and after the last. For this reason, we have only assessed the performance of predictions given predictor variable values between the boundary knots of the fitted models, as described in Section 6.1, which prevents outliers in the tails from causing extreme squared prediction error values that distort the performance measure. The same is not the case for BIC scores. In our context, BIC scores are used for model selection, including models not stemming from RCS regression. Thus, we choose to compute BIC scores without customizing specifically for RCS regression models.

Lastly, we briefly discuss model selection bias (10, pp. 58–60). The knot selection process presented in this paper uses a backward method that assesses many models. Nevertheless, in the end, only the best final models from the knot selection processes are compared in the experiments. It could be that the BIC score systematically is more favorable for one selection process than another for unknown reasons. However, in the simulation experiments, the primary performance measure and hypothesis testing are not based on BIC but on the ground truth and predictions for t=2,000 previously unseen observations for each m=1,000 finished models per knot selection process. Therefore, the estimated performance measures should not be affected by selection bias.

## 9. Related work

Several advanced methods exist for spline regression. A prominent example is penalized B-splines (P-spline) (23, 24), where smoothing splines is a specialized case (16, pp. 151–153). It is a flexible framework where splines are built from the sum of basis curves scaled by coefficients, most commonly a high number of equally distanced B-splines. The P-spline method prefers an abundance of knots and control overfitting by a roughness penalty (regularization) that smoothens or dampens the wiggliness of the curve instead of reducing the complexity of the model by removing knots. By setting coefficients to zero, certain B-splines in the mixture can, in effect, be removed. Although superficially similar to RCS regression, B-splines and P-splines are different methods from RCS regression. The knot selection presented in this paper concerns the latter.

Other techniques adaptively place knots or choose spline basis functions. Typically, these advanced methods produce relatively complex models or target slightly different problems, such as hybrid adaptive splines (25) for when there is an underlying function that is spatially inhomogeneous in its degree of complexity. In comparison, restricted cubic splines can easily be used to include non-linear relationships in a wide variety of models (6).

## 10. Conclusion

We have presented a knot selection process and greedy state space search algorithm for RCS regression and implemented it as part of an open-source R-package, knutar. The example simulation experiments show lower prediction errors and improved goodness of fit compared to placing an equivalent number of inner knots by a regular sequence of quantiles. The presented knot selection process can be used as an alternative to the standard process when the curve approximation is challenging due to several critical points, regions where the predictor variable’s observations are sparse, or both. It can also reduce overfitting in the more densely distributed regions of the predictor variable observations.

## Data Availability

The datasets presented in this study can be found in online repositories. The names of the repository/repositories can be found in the article, and the the dataset used for the experiments can be found in the Supplementary Material.
